# Supplementary material for: A randomized controlled trial for response of microbiome network to exercise and diet intervention in patients with nonalcoholic fatty liver disease
Source: Nat Commun. 2022 May 10;13:2555. doi: 10.1038/s41467-022-29968-0 (PMC9091228; doi:10.1038/s41467-022-29968-0)
Supplement: Supplementary file 1 — Supplementary Information [file 41467_2022_29968_MOESM1_ESM.pdf]

Supplementary Information

Supplementary figures

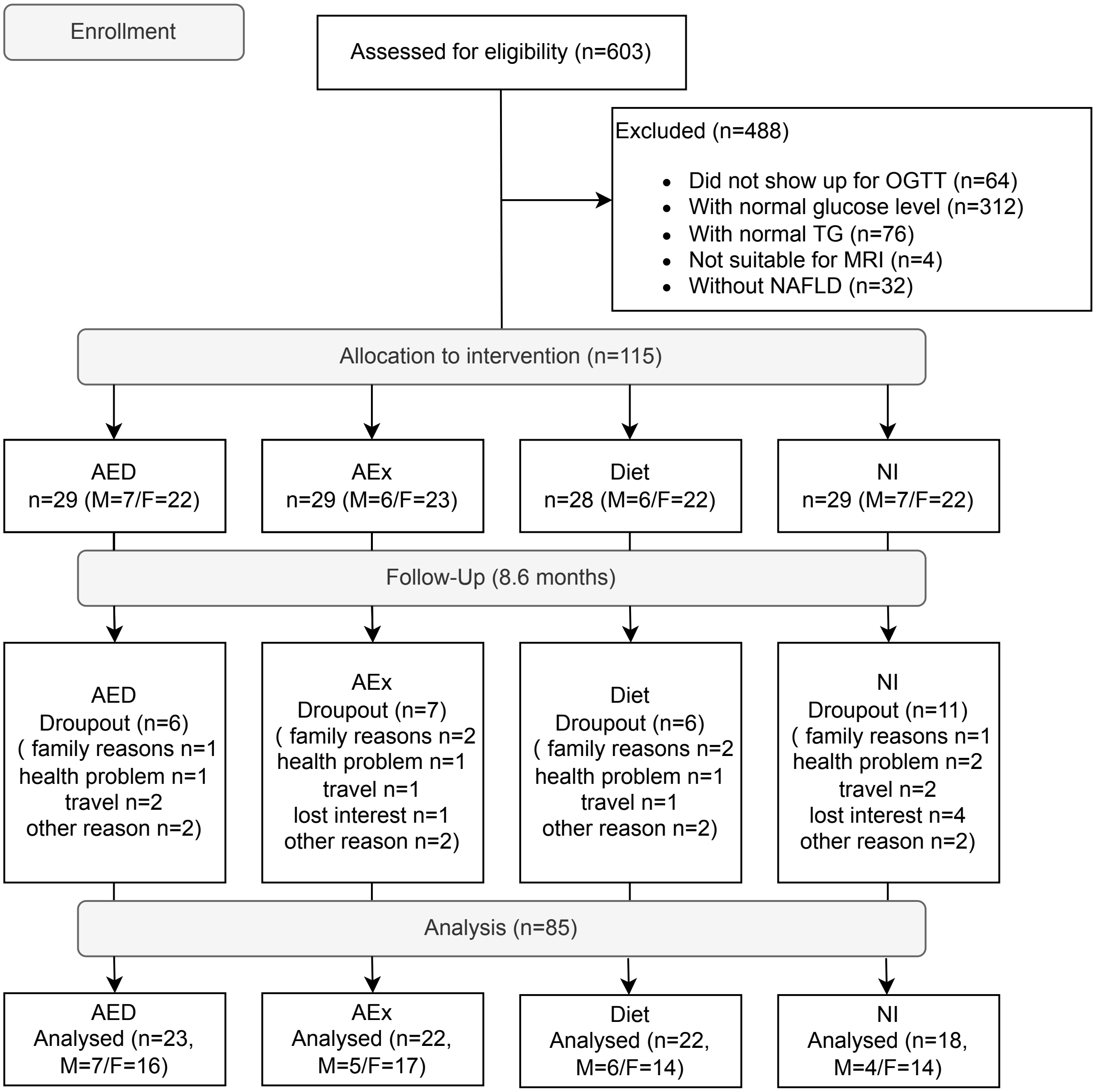

Fig. S1 CONSORT diagram. OGTT = oral glucose tolerance test; TG = triglyceride; MRS = proton magnetic resonance spectroscopy; M = male; F = female.

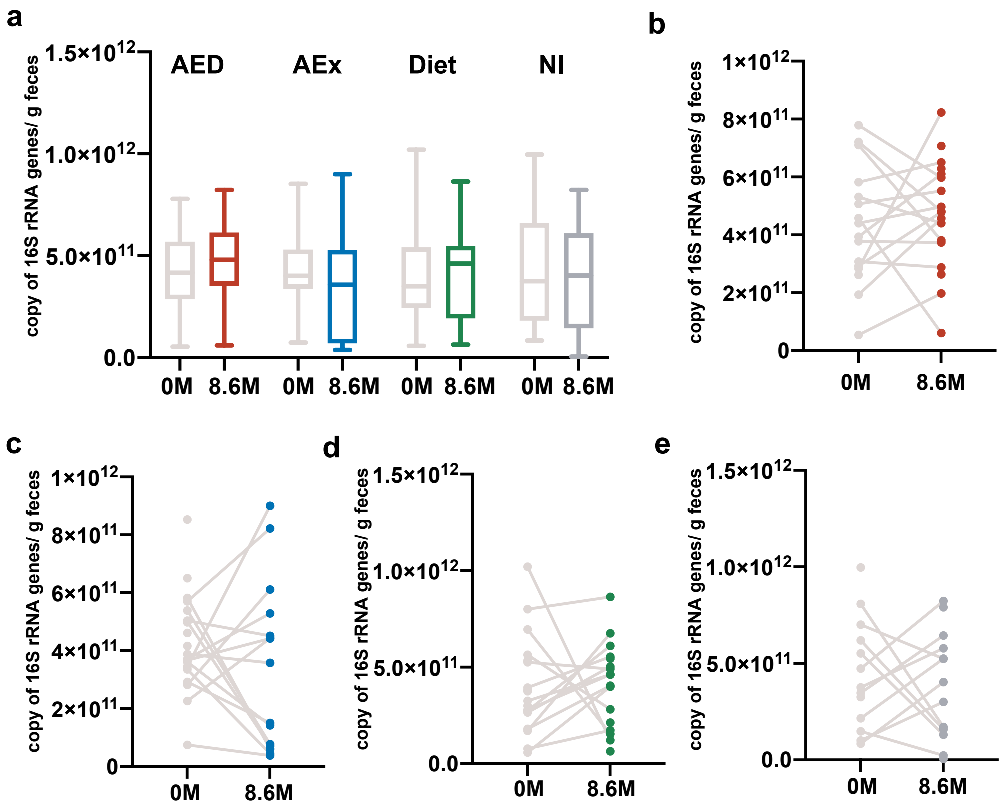

Fig. S2 Changes in the absolute abundance of gut microbiota before and after intervention. (**a**) Box plot of microbiome absolute abundance in four groups at two time points. No significant differences were observed between the groups before and after intervention. The p-values at baseline and 8.6 months were 0.96 and 0.50, respectively. Data were expressed as mean ± SD. Comparisons of change between groups were performed using one-way analysis of variance (ANOVA). (**b-e**) There are no significant absolute abundance changes within the groups after intervention (two-tailed paired test. b: AED: $n = 20$, $p = 0.79$; c: AEx: $n = 20$, $p = 0.63$; d: Diet: $n = 21$, $p = 0.72$; e: NI: $n = 15$, $p = 0.91$).

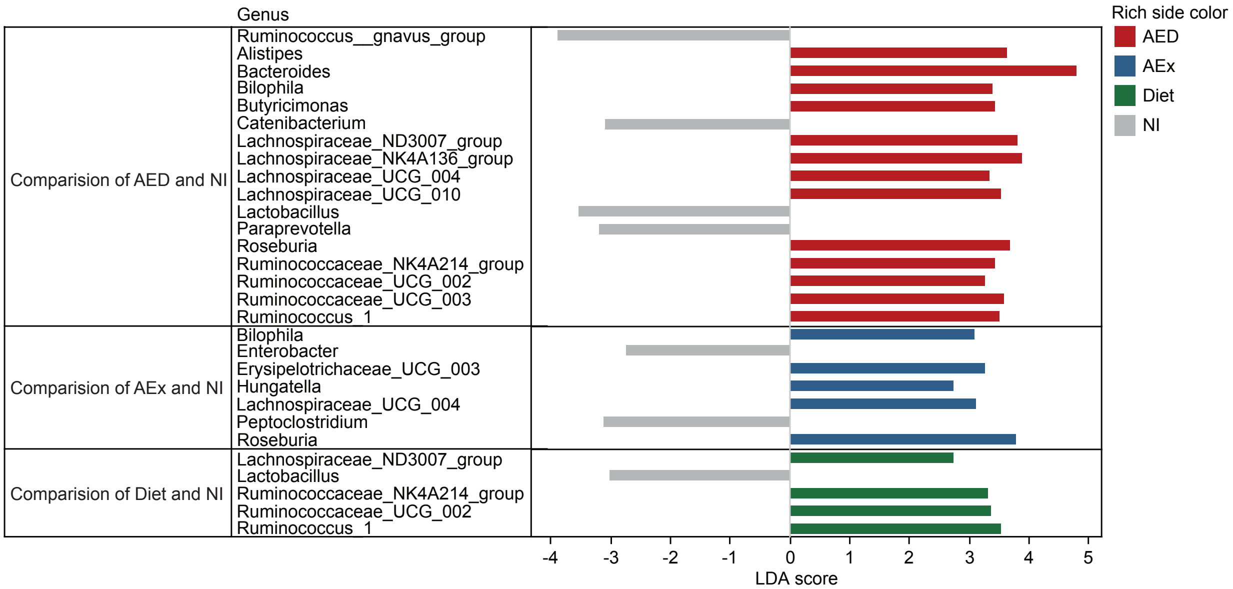

Fig. S3 Differential genus between the AED/AEx/Diet groups and NI group. LEfSe analysis in genus level (adjusted $p < 0.05$, log2 fold change > 2).

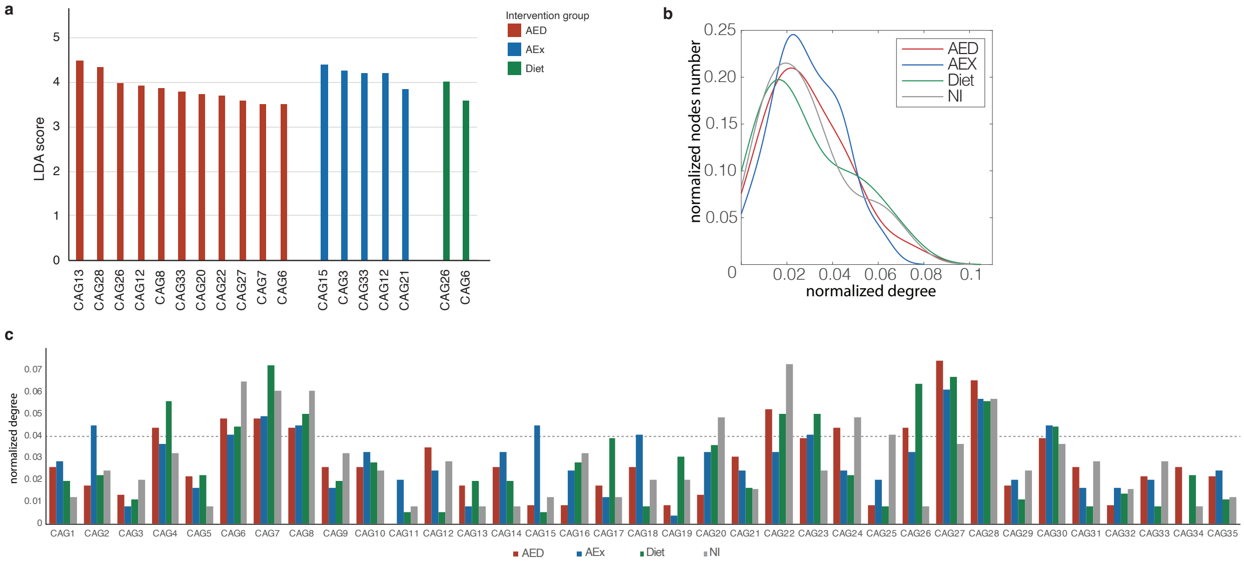

Fig. S4 CAG abundance and co-occurrence network features. (**a**) Differences in CAGs between AED/AEx/Diet groups and the NI group after intervention. CAGs: co-abundance groups. LEfSe analysis was performed under the condition of adjusted $p < 0.05$ and log2 fold change > 2. (**b**) The distribution of the CAG nodes corresponding to different degrees. (**c**) Normalized degree of each CAG node. AED: n = 20, AEx: n = 20, Diet: n = 21, NI: n = 15.

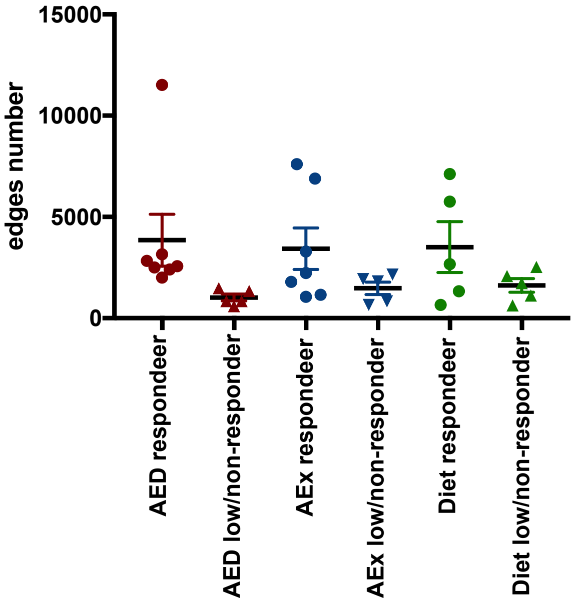

Fig. S5 Relative changes of the Singel SparCC network edge number. Data were expressed as mean ± SEM. Significance was calculated by two-tailed unpaired t test. No statistical significance was observed within groups (AED: n = 12, FC of responder and low/non-responder = 3.0, p = 0.078; AEx: n = 12, FC = 2.3, p = 0.15; Diet: n = 10, FC = 2.2, p = 0.18). Dots with different colors represent subjects.

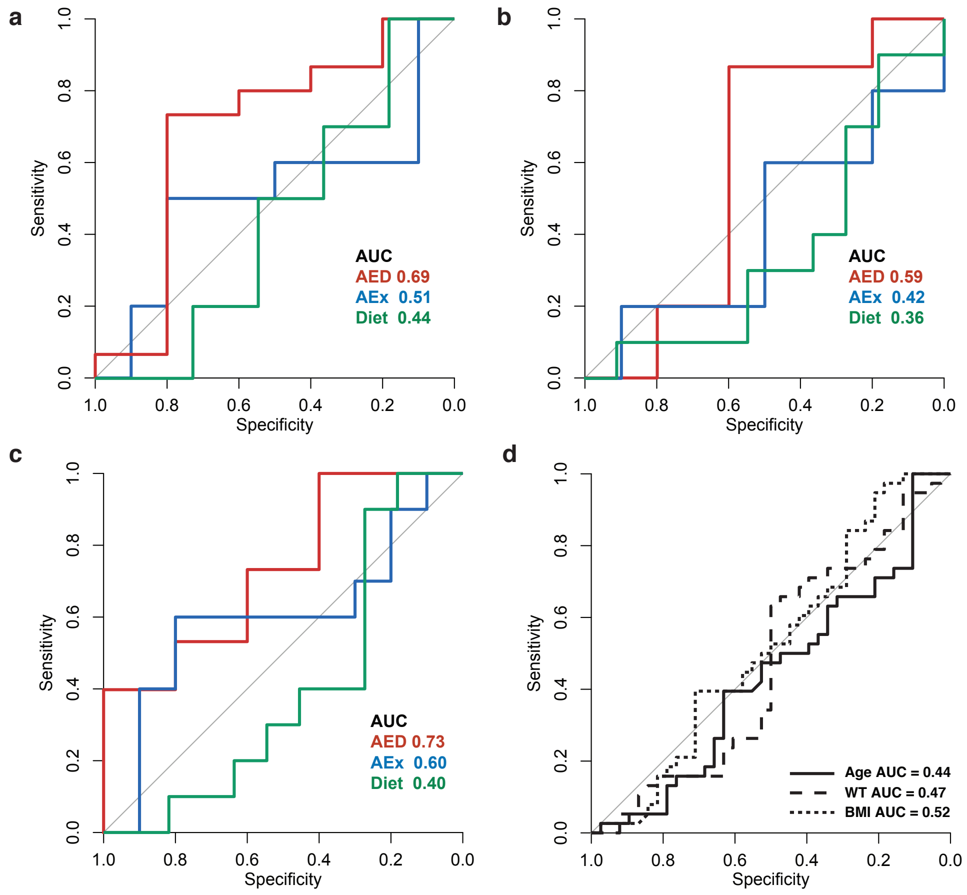

Fig. S6 The ROC curve of physical parameters predicting the effect of intervention response. (**a-c**) AED: Arobic exercise + diet; AEx: Arobic exercise and Diet groups. Age as pridictor (**a**), Body weight as prodoctor (WT, **b**) and body mass index as predictor (BMI, **c**) respectively. (**d**) all intervention groups together. AED: n = 12, AEx: n = 12, Diet: n = 10.

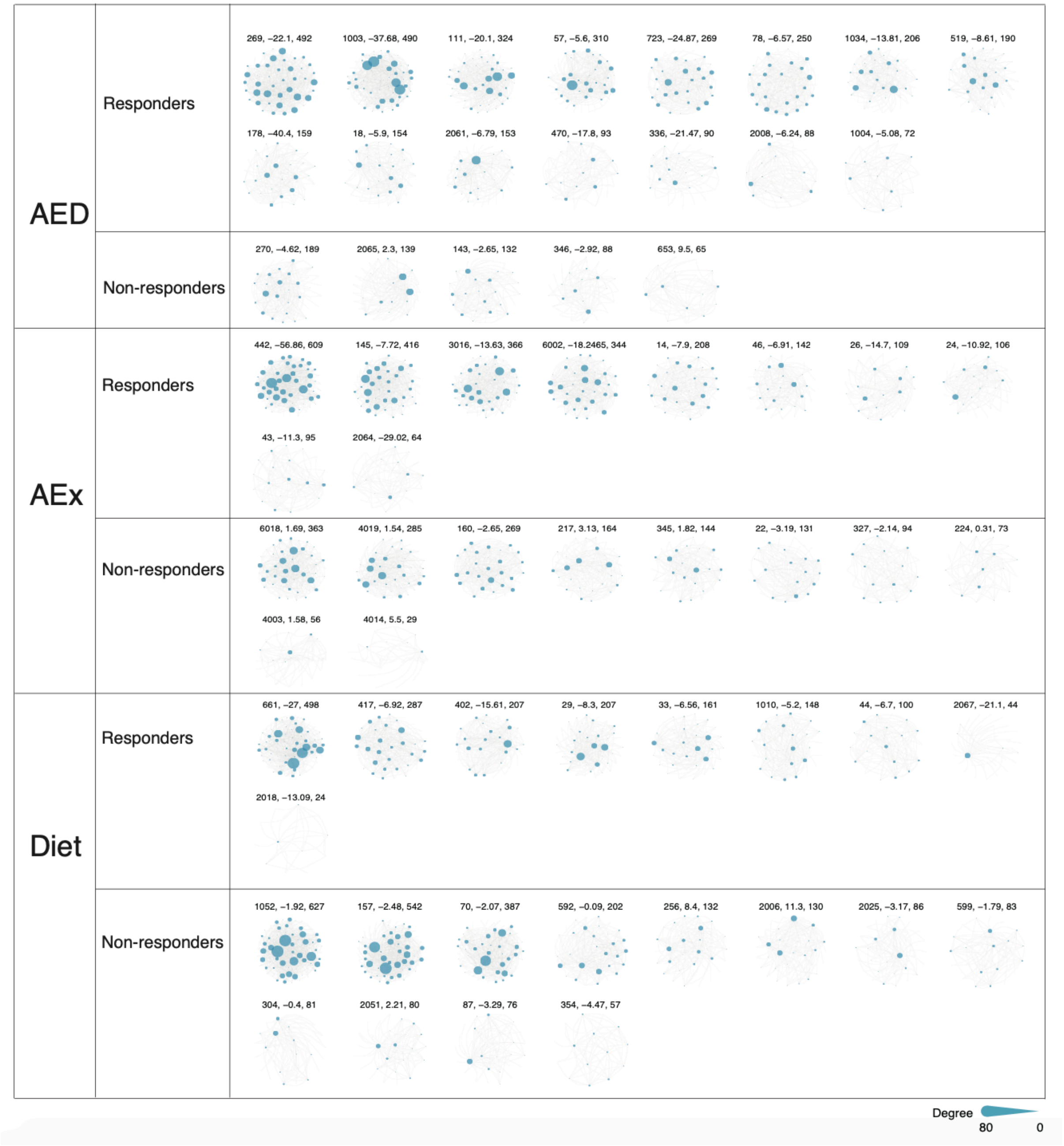

Fig. S7 The Single SparCC network for each individual before intervention in AED/AEx/Diet groups based on 16s rRNA sequence data. Three numbers under every individual network represent subject ID, HFC percentage change and edge numbers respectively.

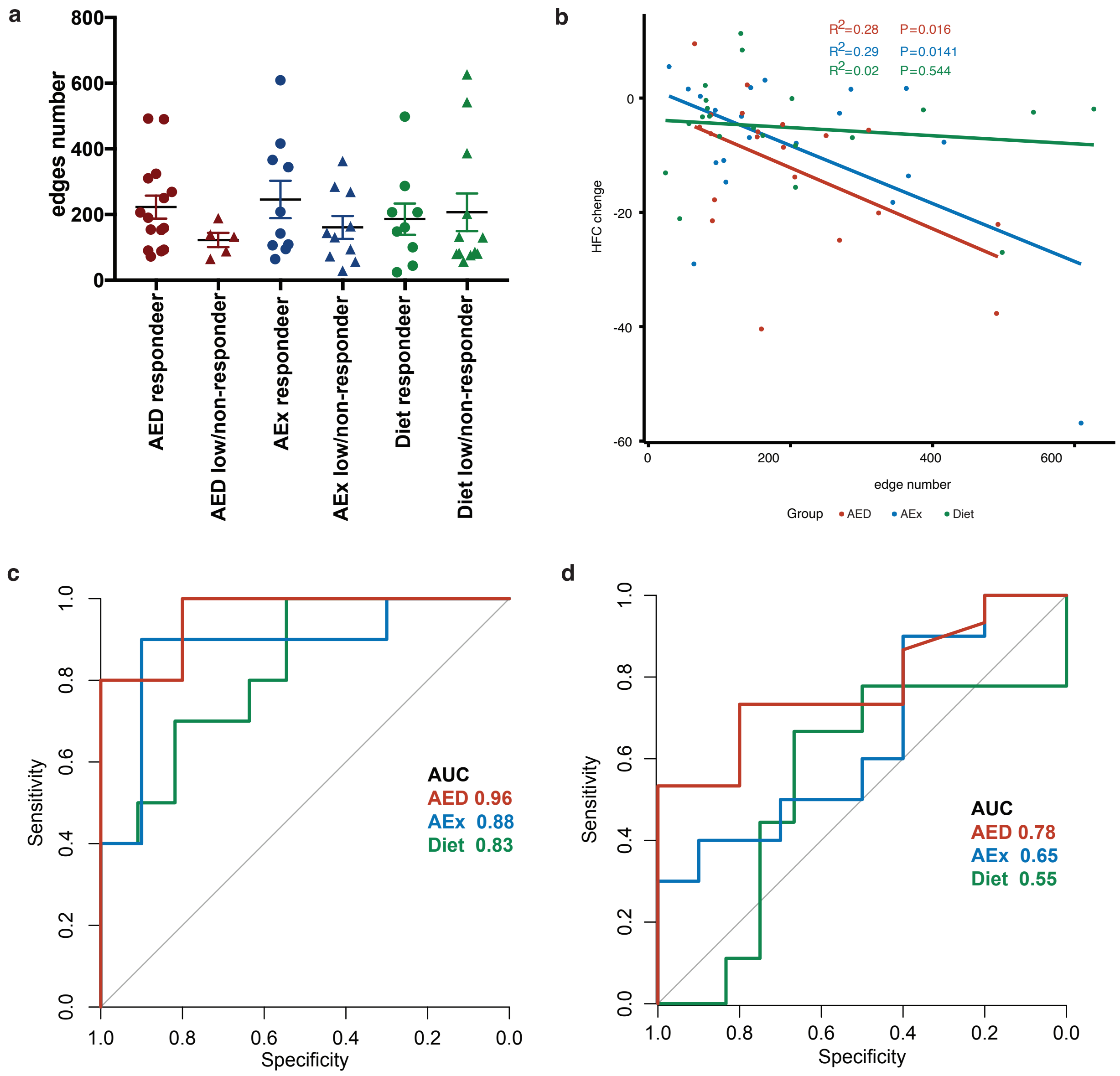

Fig. S8 Intervention efficiency predicted by individual baseline gut microbial networks on 16s rRNA sequence data. (**a**) Relative changes of the Single SparCC network edge number. Data were expressed as mean ± SEM. Significance was calculated by two-tailed unpaired t test. No statistical significance was observed within groups (AED: FC of responder and low/non-responder = 1.8, p = 0.13; AEx: FC = 1.5, p = 0.22; Diet: FC = 0.90, p = 0.79). Dots with different colors represent subjects. (**b**) Linear regressions of edge number (Single SparCC network) with the change of HFC after intervention. (**c**) ROC curve by supervised Lasso model performance. (**d**) ROC curve of edge number for differential responders from low/non-responders within each intervention group by unsupervised classification. AED: n = 20, AEx: n = 20, Diet: n = 21, NI: n = 15.

Supplementary table

Table S1. Comparison of baseline to the follow-up values of body composition and clinical biomarkers controlled for the baseline value and intervention duration.

Variables		NI (n=18)	AEx (n=22)	Diet (n=22)	AED (n=23)	Group by time	
	0-m§	8.6-m	8.6-m	8.6-m	8.6-m	*p*	Partial η2
Age (years)¶		60.6 (59.0 – 62.2)	59.3 (57.3 – 61.3)	59.7 (57.7 – 61.7)	59.4 (57.8 – 61.1)	0.765	
Height (cm)¶		156 (151 – 161)	161 (157 – 166)	160 (158 – 163)	164 (161 – 168)**	0.033	
HFC (%)	18.3	16.1 (12.4 – 19.9)	10.3 (7.1 – 13.5)*	12.9 (9.8 – 16.1)†	7.34 (4.1 – 10.6)**	0.005	0.167
Weight (kg)	68.9	68.6 (67.4 – 69.7)	67.2 (66.3 – 68.2)	67.7 (66.8 – 68.6)	67.2 (66.2 – 68.1)	0.240	0.058
Fat (%)	37.1	37.9 (37.1 – 38.6)	35.8 (35.2 – 36.5)***	36.6 (36.0 – 37.2)*	36.5 (35.8 – 37.1)**	0.002	0.193
BMI	26.6	26.4 (26.0 – 26.9)	25.9 (25.5 – 26.3)	26.1 (25.8 – 26.5)	26.0 (25.6 – 26.4)	0.321	0.050
Glucose (mmol/L)	5.58	5.89 (5.57 – 6.22)	5.43 (5.15 – 5.71)	5.81 (5.53 – 6.08)	5.78 (5.50 – 6.06)	0.128	0.077
Insulin (µIU/mL)	16.4	13.4 (10.4 – 16.3)	15.7 (13.2 – 18.3)	14.7 (12.2 – 17.2)	16.0 (13.4 – 18.6)	0.554	0.029
HBA1c (%)	6.14	6.11 (5.94 – 6.29)	6.12 (5.97 – 6.27)	6.09 (5.95 – 6.24)	6.03 (5.88 – 6.18)	0.857	0.011
HOMA-IR	4.11	3.55 (2.72 – 4.38)	3.89 (3.16 – 4.61)	3.80 (3.09 – 4.51)	4.10 (3.38 – 4.82)	0.799	0.014
Triglyceride (mmol/L)	2.19	2.09 (1.45 – 2.73)	2.24 (1.69 – 2.79)	1.78 (1.25 – 2.32)	2.49 (1.94 – 3.04)	0.329	0.048
FFA (mmol/L)	0.575	0.47 (0.37 – 0.56)	0.36 (0.28 – 0.44)	0.46 (0.39 – 0.53)	0.47 (0.39 – 0.55)	0.158	0.075
ALT (U/L)	23.9	29.9 (24.3 – 35.5)	20.5 (15.6 – 25.3)*	17.2 (12.5 – 21.9)**	21.2 (16.3 – 26.0)*	0.010	0.149
AST (U/L)	24.6	26.6 (22.0 – 31.2)	24.6 (20.8 – 28.6)	22.6 (18.8 – 26.4)	22.1 (18.2 – 26.0)	0.428	0.040
GGT (U/L)	26.9	26.3 (19.6 – 32.9)	30.3 (24.8 – 35.8)	27.2 (21.6 – 32.8)	30.9 (25.4 – 36.4)	0.625	0.025

Subjects were those who have both baseline and follow-up gut microbiota data (Marginal mean (95% confidence interval)). General linear model of analysis of variance controlled for covariates (ANCOVA) for repeated measures (2 factor interactions: group x time) and controlled for baseline value of each variable and intervention duration followed by Sidak correction for multiple comparison between the groups. Contrast results (K Matrix) were used to localize the group differences: * $p < 0.05$, ** $p < 0.01$ and *** $p < 0.001$ by contract to the NI group. † $p < 0.05$ by contract to the AED group. Partial η2 = effect size. § Baseline marginal means are the same for all groups since values were controlled for baseline value of each variable and intervention duration. NI = No intervention group; AEx = Exercise group; AED = AEx+Diet group; HFC = Hepatic fat content; HBA1c = glycated haemoglobin; HOMA-IR = homeostasis model assessment of insulin resistance index, FFA = Free fatty acids, ALT = Alanine

aminotransferase; AST = aspartate aminotransferase; GGT = γ-glutamyltransferase. All the biochemical markers are fasting samples.

Table S2. The core ASV of CAG at different taxonomic levels.

CAG	Core ASV	phylum	classs	order	family	genus
CAG1	ASV2	Proteobacteria	Gammaproteobacteria	Enterobacteriales	Enterobacteriaceae	*Escherichia-Shigella*
CAG2	ASV37	Firmicutes	Clostridia	Clostridiales	Lachnospiraceae	*Anaerostipes*
CAG3	ASV4	Firmicutes	Clostridia	Clostridiales	Ruminococcaceae	*Faecalibacterium*
CAG4	ASV5	Firmicutes	Clostridia	Clostridiales	Lachnospiraceae	*Blautia*
CAG5	ASV24	Firmicutes	Bacilli	Lactobacillales	Streptococcaceae	*Streptococcus*
CAG6	ASV101	Bacteroidetes	Bacteroidia	Bacteroidales	Bacteroidaceae	*Bacteroides*
CAG7	ASV233	Firmicutes	Clostridia	Clostridiales	Ruminococcaceae	*Ruminococcaceae UCG-4*
CAG8	ASV70	Bacteroidetes	Bacteroidia	Bacteroidales	Rikenellaceae	*Alistipes*
CAG9	ASV21	Bacteroidetes	Bacteroidia	Bacteroidales	Bacteroidaceae	*Bacteroides*
CAG10	ASV99	Firmicutes	Erysipelotrichia	Erysipelotrichales	Erysipelotrichaceae	*Erysipelatoclostridium*
CAG11	ASV38	Bacteroidetes	Bacteroidia	Bacteroidales	Bacteroidaceae	*Bacteroides*
CAG12	ASV17	Bacteroidetes	Bacteroidia	Bacteroidales	Bacteroidaceae	*Bacteroides*
CAG13	ASV7	Firmicutes	Negativicutes	Selenomonadales	Acidaminococcaceae	*Phascolarctobacterium*
CAG14	ASV36	Bacteroidetes	Bacteroidia	Bacteroidales	Prevotellaceae	*Prevotella 9*
CAG15	ASV92	Bacteroidetes	Bacteroidia	Bacteroidales	Prevotellaceae	*Prevotella 9*
CAG16	ASV63	Bacteroidetes	Bacteroidia	Bacteroidales	Prevotellaceae	*Prevotella 9*
CAG17	ASV94	Bacteroidetes	Bacteroidia	Bacteroidales	Tannerellaceae	*Parabacteroides*
CAG17	ASV194	Bacteroidetes	Bacteroidia	Bacteroidales	Tannerellaceae	*Parabacteroides*
CAG18	ASV3	Actinobacteria	Actinobacteria	Bifidobacteriales	Bifidobacteriaceae	*Bifidobacterium*
CAG19	ASV12	Bacteroidetes	Bacteroidia	Bacteroidales	Bacteroidaceae	*Bacteroides*
CAG20	ASV52	Bacteroidetes	Bacteroidia	Bacteroidales	Bacteroidaceae	*Bacteroides*
CAG21	ASV49	Bacteroidetes	Bacteroidia	Bacteroidales	Bacteroidaceae	*Bacteroides*
CAG22	ASV89	Firmicutes	Clostridia	Clostridiales	Ruminococcaceae	*Ruminococcaceae NK4A214 group*
CAG23	ASV102	Firmicutes	Clostridia	Clostridiales	Christensenellaceae	*Christensenellaceae R-7 group*
CAG24	ASV141	Actinobacteria	Coriobacteriia	Coriobacteriales	Coriobacteriaceae	*Collinsella*
CAG25	ASV46	Bacteroidetes	Bacteroidia	Bacteroidales	Bacteroidaceae	*Bacteroides*
CAG26	ASV45	Bacteroidetes	Bacteroidia	Bacteroidales	Bacteroidaceae	*Bacteroides*

CAG27	ASV106	Firmicutes	Clostridia	Clostridiales	Lachnospiraceae	*Coprococcus 2*
CAG28	ASV11	Firmicutes	Clostridia	Clostridiales	Ruminococcaceae	*Subdoligranulum*
CAG29	ASV23	Firmicutes	Clostridia	Clostridiales	Lachnospiraceae	*Roseburia*
CAG30	ASV1	Firmicutes	Clostridia	Clostridiales	Ruminococcaceae	*Faecalibacterium*
CAG31	ASV32	Firmicutes	Negativicutes	Selenomonadales	Veillonellaceae	*Dialister*
CAG32	ASV6	Firmicutes	Clostridia	Clostridiales	Lachnospiraceae	*Agathobacter*
CAG33	ASV62	Proteobacteria	Gammaproteobacteria	Betaproteobacteriales	Burkholderiaceae	*Parasutterella*
CAG34	ASV30	Firmicutes	Clostridia	Clostridiales	Ruminococcaceae	*Ruminococcus 2*
CAG35	ASV25	Firmicutes	Erysipelotrichia	Erysipelotrichales	Erysipelotrichaceae	*Erysipelotrichaceae UCG-3*
