# Supplementary material for: A randomized controlled trial for response of microbiome network to exercise and diet intervention in patients with nonalcoholic fatty liver disease
Source: Nat Commun. 2022 May 10;13:2555. doi: 10.1038/s41467-022-29968-0 (PMC9091228; doi:10.1038/s41467-022-29968-0)
Supplement: Supplementary file 3 — Reporting Summary [file 41467_2022_29968_MOESM3_ESM.pdf]

Corresponding author(s): Sulin Cheng

Last updated by author(s): Mar 4, 2022

Reporting Summary

Nature Research wishes to improve the reproducibility of the work that we publish. This form provides structure for consistency and transparency in reporting. For further information on Nature Research policies, see our Editorial Policies and the Editorial Policy Checklist.

Statistics

For all statistical analyses, confirm that the following items are present in the figure legend, table legend, main text, or Methods section.

n/a	Confirmed	
☐	☒	The exact sample size (*n*) for each experimental group/condition, given as a discrete number and unit of measurement
☐	☒	A statement on whether measurements were taken from distinct samples or whether the same sample was measured repeatedly
☐	☒	The statistical test(s) used AND whether they are one- or two-sided<br>*Only common tests should be described solely by name; describe more complex techniques in the Methods section.*
☐	☒	A description of all covariates tested
☐	☒	A description of any assumptions or corrections, such as tests of normality and adjustment for multiple comparisons
☐	☒	A full description of the statistical parameters including central tendency (e.g. means) or other basic estimates (e.g. regression coefficient) AND variation (e.g. standard deviation) or associated estimates of uncertainty (e.g. confidence intervals)
☐	☒	For null hypothesis testing, the test statistic (e.g. *F*, *t*, *r*) with confidence intervals, effect sizes, degrees of freedom and *P* value noted<br>*Give P values as exact values whenever suitable.*
☒	☐	For Bayesian analysis, information on the choice of priors and Markov chain Monte Carlo settings
☐	☒	For hierarchical and complex designs, identification of the appropriate level for tests and full reporting of outcomes
☐	☒	Estimates of effect sizes (e.g. Cohen's *d*, Pearson's *r*), indicating how they were calculated

Our web collection on statistics for biologists contains articles on many of the points above.

Software and code

Policy information about availability of computer code

Data collection	No software was used to collect the biological samples.
Data analysis	QIIME 2 (https://qiime2.org)<br>LEfSe (http://huttenhower.sph.harvard.edu/galaxy/)<br>MATLAB (version R2020a)<br>R (version 4.0)<br>Cytoscape (version 3.8.1)

For manuscripts utilizing custom algorithms or software that are central to the research but not yet described in published literature, software must be made available to editors and reviewers. We strongly encourage code deposition in a community repository (e.g. GitHub). See the Nature Research guidelines for submitting code & software for further information.

Data

Policy information about availability of data

All manuscripts must include a data availability statement. This statement should provide the following information, where applicable:

- Accession codes, unique identifiers, or web links for publicly available datasets
- A list of figures that have associated raw data
- A description of any restrictions on data availability

The raw Illumina sequence data including 16s rRNA sequence and metagenomics generated in this study have been deposited to the NCBI database under BioProject accession no. PRJNA757939 (https://www.ncbi.nlm.nih.gov/bioproject/?term=PRJNA757939). All other data that support the findings of this study are available from the corresponding author upon reasonable request. The analysed data in this study are provided in the Source Data file.
Figure 2, 3, 4, and 5 are associated with raw data.

Field-specific reporting

Please select the one below that is the best fit for your research. If you are not sure, read the appropriate sections before making your selection.

☒ Life sciences ☐ Behavioural & social sciences ☐ Ecological, evolutionary & environmental sciences

For a reference copy of the document with all sections, see nature.com/documents/nr-reporting-summary-flat.pdf

Life sciences study design

All studies must disclose on these points even when the disclosure is negative.

Sample size

In the initial study design, we estimated the sample size based on previous literature of liver fat and microbiota as primary outcome variable. We estimated that 34 subjects in each group would have 85% power for mean comparison between the groups and therefore we attempted targeting to have 50 subjects in each group. However, during the recruitment period, we found that only about 20% of the subjects met the inclusion criteria and due to the limited funding, we re-calculated the sample size based on of our previous similar type of study result (changing in fat mass) (doi:10.1016/j.sleep.2016.02.010, doi:10.3390/nu8110751). Thus, for this report, sample size estimation for the primary outcome HFC with 29 individuals has 95% power to test against the hypothesis that there is change in any group. Further, when intervention groups compared with NI group having 17 subjects, the power for the HFC was 84% with <0.05 two-sided significance level. After every subject reached minimal 6 months intervention, the study did not continue to follow-up all the subjects and consequently, the follow-up study was terminated.

Data exclusions

Body mass index (BMI) > 38 kg/m2; serious cardiovascular or musculoskeletal problems; diagnosed Type 1 diabetes and T2D; and mental illness.

Replication

The results of the stability of the co-occurrence network and the characteristics of individual gut microbial network assessed by 16S sequencing was replicated by Metagenomic sequencing. However, due to the different sequencing depths of the two methods and the sample of metagenomic sequencing was a subset of 16S sequencing, differential taxa analysis results could not be able to replicate. As we know, the two kinds of sequencing data cannot be directly compared due to the 16S rRNA genes only account for a small percentage of the whole genome. Therefore, there are very few genes found in16S rRNA as in the shotgun metagenomics data.

Randomization

This study was a randomized controlled trial. Of the 603 eligible subjects, 115 met the inclusion criteria and were randomly assigned (1:1:1:1) to aerobic exercise (AEx, n = 29), diet intervention (Diet, n = 28), aerobic exercise plus diet intervention (AED = 29), or no intervention (NI = 29) groups for minimal 6 months. A computer program was used to generate the block randomization sequence (block size 20) and was controlled by a researcher not involved in the selection of the participants.

Blinding

Although lifestyle interventions cannot be performed in a double-blinded fashion (since study subjects clearly are aware of the type of intervention), investigators were blinded for the tests and analyses during the entire study.

Reporting for specific materials, systems and methods

We require information from authors about some types of materials, experimental systems and methods used in many studies. Here, indicate whether each material, system or method listed is relevant to your study. If you are not sure if a list item applies to your research, read the appropriate section before selecting a response.

Materials & experimental systems

n/a	Involved in the study	
☒	☐	Antibodies
☒	☐	Eukaryotic cell lines
☒	☐	Palaeontology and archaeology
☒	☐	Animals and other organisms
☐	☒	Human research participants
☐	☒	Clinical data
☒	☐	Dual use research of concern

Methods

n/a	Involved in the study	
☒	☐	ChIP-seq
☒	☐	Flow cytometry
☒	☐	MRI-based neuroimaging

Human research participants

Policy information about studies involving human research participants

Population characteristics

Men or women aged 50–65 years with fasting glucose between 5.6 to 6.9 mmol/or glucose between 7.8 to 11.0 mmol/L 2 hour after the intake of glucose (75 g), diagnosed as NAFLD by 1H MRS (liver fat >5%) and by questionnaire that on-going or recent alcohol consumption is <21 drinks on average per week in men and < 14 drinks on average per week in women; no chronic cardiovascular, serious musculoskeletal or gastrointestinal problems and not on extreme diets; and for women, serum follicle-stimulating hormone level greater than 30 IU/L and last menstruation more than 6 months ago but within 10 years.

Recruitment	Participants were recruited from 7 health clinical service centers in the Shanghai Yangpu district. Potential subjects were selected from the outpatient pool with both a liver ultrasound (for diagnosing fatty liver) and fasting blood results (for diagnosing diabetes) on file during the period of 2012 to 2013. After signing the screening consent, patients were asked to fill in a screening questionnaire to assess their health and medication history, including physical activity and alcohol consumption, and to undergo a glucose tolerance test. After this stage, there were 603 eligible subjects. Those subjects who met the inclusion criteria and were willing to participate in this study were invited for measurements of height, weight, waist circumference, blood pressure, physical performance, and body composition by dual-X-ray densitometry (DXA, GE, USA, Prodigy). They were also invited for a proton magnetic resonance spectroscopy (1H MRS) scan for non-invasive assessment of liver fat, with NAFLD defined as >5% liver fat content (Siemens Magnetom Verio 3 T, Siemens AG, Erlangen, Germany). The dietary and exercise intervention methods are often different from trial to trial, and even if the intervention methods are the same, the intervention contents and intensity are different which makes the comparison with other trials challenging.
Ethics oversight	The study was approved by the Ethics Committee of Shanghai Institute of Nutrition (06.01.2013, ref: 2013-003) and has been registered in the International Standard Randomized Controlled Trial Number Register (ISRCTN 42622771). All participants provided written informed consent prior to the study. The study was followed the world medical association declaration of Helsinki – Ethical principles for medical research involving human subjects.

Note that full information on the approval of the study protocol must also be provided in the manuscript.

Clinical data

Policy information about clinical studies

All manuscripts should comply with the ICMJE guidelines for publication of clinical research and a completed CONSORT checklist must be included with all submissions.

Clinical trial registration	The International Standard Randomized Controlled Trial Number Register (ISRCTN 42622771)
Study protocol	ISRCTN42622771
Data collection	The laboratory tests and interventions were performed and managed by the research team from Shanghai University of Sport, Shanghai Jiao Tong University, Shanghai Yangpu District Health Care Service Centres and Yungpu Shidong Hospital, and Clinical Nutrition Centre at Fudan University Huadong Hospital, Shanghai, China.<br>Data collection was performed during January 2013 to December 2015.
Outcomes	Primary outcomes:<br>1. Glucose tolerance test results, insulin and triglycerides<br>2. Liver fat content % (1H MRS)<br>3. Gut microbiota compositions (16S rRNA and metagenomics sequencing)<br>The measurements for primary outcomes were assessed at the baseline and 6-month time points.<br>Secondary outcomes:<br>1. Questionnaires of lifestyle, behavioral characteristics, diet, physical activity, health condition<br>2. Anthropometry (height, body weight)<br>3. Blood pressure<br>4. Body composition (fat mass, lean mass and bone mass)<br>5. Physical fitness and heart rate (UKK walk test)<br>6. Muscle strength (maximal isometric voluntary contraction of the right grip, left elbow flexors and left knee extensors)<br>7. Venous blood sample (total cholesterol, HDL-cholesterol, triglycerides, LDL-cholesterol, apolipoproteins A-I and B, lipoprotein (a), cholesterylester fatty acid composition, free fatty acid profile and sex hormones, inflammation biomarkers).
